# Associations of effort-reward imbalance at work and quality of life among workers after stroke: a one-year longitudinal study in Thailand

**DOI:** 10.1186/s12889-023-16784-4

**Published:** 2023-10-03

**Authors:** Siripan Naknoi, Jian Li, Pongrama ramasoota, Xinyue Liu, Liwei Chen, Suparat Phuanukoonnon, Ngamphol Soonthornworasiri, Orawan Kaewboonchoo

**Affiliations:** 1 Department of Social and Environmental Medicine, Faculty of Tropical Medicine, Mahidol University, Bangkok, 10400 Thailand; 2 Department of Environmental Health Sciences, Fielding School of Public Health, School of Nursing, University of California Los Angeles, Los Angeles, CA 90095 USA; 3 Department of Public health Nursing, Faculty of Public health, Mahidol University, Bangkok, 10400 Thailand; 4 Department of Epidemiology, Fielding School of Public Health, University of California Los Angeles, Los Angeles, CA 90095 USA; 5 Department of Tropical Hygiene, Faculty of Tropical Medicine, Mahidol University, Bangkok, 10400 Thailand

**Keywords:** Effort-reward imbalance, Over-commitment, Quality of life, Stroke, Worker, Longitudinal study

## Abstract

Stroke incidence is increasing among working-age population, but the role of psychosocial stress in the workplace in predicting quality of life (QoL) after stroke onset is understudied. This longitudinal study aimed to investigate the relationship between work stress, measured by the effort-reward imbalance (ERI) model, and QoL over one-year period among 103 Thai workers who had experienced a stroke. The study evaluated the effort (E)-reward (R) ratio and over-commitment, the extrinsic and intrinsic components of the ERI model, before discharge; QoL was repeatedly measured at baseline, six months, and 12 months after discharge, respectively, using the Short Form Version 2 (SF-12v2) indicators of physical and mental health composite scores. Generalized estimating equations were used to examine longitudinal relationships between work stress at baseline and QoL over one year by testing the hypotheses that E-R ratio and over-commitment would have direct effects on QoL, and potential moderating effects of over-commitment on E-R ratio and QoL. The results supported the ERI model partially, as over-commitment was significantly associated with poor mental health (coefficient − 8.50; 95% CI: -13.79, -3.20) after adjusting baseline sociodemographic, behavioral, and clinical characteristics, while the E-R ratio was not significantly associated with physical or mental health; the interaction between the E-R ratio and over-commitment was also not significant. These findings suggest that more attention should be paid to workers’ personal coping skills and ability to handle work-related problems and prioritize interventions that address over-commitment to promote long-term mental health among workers with stroke.

## Introduction

Stroke is a major public health issue worldwide, causing death and long-term disability burdens, particularly among countries with middle-low incomes [1]. According to the Global Update 2019 data, stroke in young people is on the rise [2]. More than half of the stroke patients were found in people younger than 65 years, particularly the incidence of stroke in people under the age of 50 is increasing [3]. It was noted that, during the past two decades, hospitalization of first acute strokes was on a steady rise among people aged 18–54, who were economically active [4]. The major concern after a stroke among the working-age population was about a poor long-term outcomes [5], including functional disabilities and psychosocial impairments [6], core elements of individuals’ quality of life (QoL) [7–9]. In several international investigations with prospective cohort design, the importance of QoL physical and mental domains has been proven by predicting post-stroke survival [10].

Traditionally, causes of stroke are sociodemographic factors such as age and sex, vascular risk factors such as hypertension, diabetes mellitus, hyperlipemia, and behavioral risks such as current smokers, alcohol drinkers, and obesity [11]. In recent years, the role of psychosocial work environment in explaining the development of cardiovascular diseases has drawn much attention [12]. The Job Demand-Control (JDC) model, one of widely used work stress concepts which focuses on task characteristics by combining high job demand and low job control, has suggested potential links between JDC and elevated risk of stroke [13]. It is notable that, among stroke victims at working age, a couple of more recently published studies indicated a potential contribution of a complementary concept of work stress, the Effort-Reward Imbalance (ERI) model [14, 15] developed by Siegrist [16, 17]. The extrinsic component of the ERI model is defined as an imbalance between high effort (e.g., job obligations and demands) and low reward (e.g., salary, esteem, job security). Accordingly, a stressful condition due to long-term exposure to high effort/low reward situation (e.g., as ratio term quantifying the imbalance between high effort and low reward at individual level, E-R ratio) exerts negative effects on health. In addition, over-commitment, a distinct motivational pattern of people’s coping with stress at work, acts as the intrinsic component of the ERI model. Theoretically, over-commitment can directly increase risk of disease. Also, it is assumed to moderate the effect of E-R ratio on health, as well. As mentioned above, the Swedish study suggested that E-R ratio was significantly associated with higher risk of stroke by 28% [14], and another study from Spain confirmed that E-R ratio was a strong risk factor for transitory ischemic attack and minor stroke (odds ratio = 7.91), while over-commitment significantly increased the stroke risk by 30% [15].

Regarding the predictive power of work stress on disease prognosis, only two studies from Germany and China, respectively, reported longitudinal associations of ERI with QoL among workers after coronary heart disease, which is one major type of cardiovascular diseases [18, 19]. The results found that a high baseline E-R ratio was significantly related to the poor physical and mental health of QoL during follow-up over 12 months, but over-commitment was not examined in these two studies [18, 19]. Though the findings from workers after coronary heart disease may not be well applicable for those after stroke, the other main type of cardiovascular diseases, because consequences of stroke differ from those of coronary heart disease in term of severity, complexity, and diversity, we theoretically hypothesized that ERI would play an important role to predict changes in QoL among workers after stroke, i.e., E-R ratio and over-commitment could exert direct influences on longitudinal tracking of QoL, and over-commitment might modify the effect of E-R ratio on QoL. According to the literature, more than half of young stroke survivors would return to work within one year, and the majority of them returned to the same work [20], thus exposure to stressful experiences at work would continue. For those who were out of labor market after a major disease such as stroke, it has been suggested that prior work stress, as measured by ERI, significantly predicted work exit [21], and persistently affected long-term QoL afterwards [22], based on the life course perspective [23]. Therefore, in order to fill the above-mentioned research gaps, we conducted a hospital-based one-year longitudinal study among a sample of economically active stroke survivors. Specifically, this study was conducted in Thailand, a country with rapid industrialization, resulting in high psychological pressure among Thai employees [24] and increasing morbidity and mortality rates of stroke among working age population [25]. However, research evidence on work stress and cardiovascular diseases is still limited in this country [26].

## Materials and methods

### Study design and participant

In this longitudinal study, we targeted all stroke survivors who had been admitted for the first time with transient ischemic attack (TIA) or ischemic stroke (IS) to a tertiary hospital in Bangkok, Thailand. The other inclusion criteria were age between 20 and 59 years old, either male or female, regular paid job before the stroke onset, and ability to communicate and complete surveys. The patients were diagnosed by neurologists using ICD 10 coding of stroke [27]. Prior to the data collection, a sample size of 126 subjects was defined by applying established methods for studies with repeated measures [28]. The data collection period of this study ranged from December 2020 to August 2022. Given the enormous difficulties due to lock-down and social distancing policies during the COVID-19 pandemic, 103 patients were finally recruited. The sociodemographic, behavioral, clinical information, and data on ERI were collected during hospitalization before discharge (baseline survey), QoL was collected at each follow-up assessment (baseline surveys, six-month, and 12-month follow-up surveys) via face-to-face interview. The participants selection procedure was coordinated by the researcher (S.N.) with heads of stroke units in the hospital, under permission from the patients’ neurologist or neurosurgeon, in accordance with the inclusion criteria. Before we began collecting data, all of the participants provided informed consent. The project was approved by the Ethics Committee of the Faculty of Tropical Medicine, Mahidol University (MUTM 2020-066-01).

### Collection of Sociodemographic and clinical information

A questionnaire was used to collect sociodemographic data, including age, sex, marital status, monthly income, and education level. Behavioral factors include body mass index (BMI) (kg/m^2^), current smoker, and alcohol drinker, which were gathered from interviews. And clinical characteristics data were extracted from medical records, including family history of stroke, the National Institutes of Health Stroke Scale (NIHSS) score at admission, and cardiometabolic diseases (including hypertension, diabetes mellitus, dyslipidemia) at baseline.

### Assessment of Work stress

Work stress was assessed by the Thai version of the Effort-Reward Imbalance Questionnaire (Thai ERIQ) [29]. This version was translated and back-translated from the original version, and was proven with satisfactory reliability and validity [17]. The ERI questionnaire contains three scales with 23 items, including “effort” (six items), “rewards” (eleven items), and “over-commitment” (six items). The E-R ratio was calculated by sum scores between “effort” scale and “reward” scale by weighting the items numbers of respective scales. Stressful experiences at work were quantified by two binary measures of both E-R ratio (> 1 vs. ≤ 1) and over-commitment (> upper tertile score 15 vs. ≤ 15). The Cronbach’s alpha coefficients for effort, reward, and over-commitment in this study were 0.88, 0.67, and 0.82, respectively.

### Evaluation outcome: quality of life

Quality of life (QoL) was evaluated by the Thai Short Form-12 questionnaire (SF-12v2) which has been used to assess health-related QoL in general Thai populations, including stroke patients [30, 31]. The SF-12 was developed by testing and re-testing the original Short Form-36. The SF-12 questionnaire was divided into two components: physical health (PCS: items 1, 2, 3, 4, 5, and 8) and mental health (MCS: items 6, 7, 9, 10, 11, and 12). With multiple scales consisting of eight domains: physical function (2 items), role physical (2 items), bodily pain (1 item), general health (1 item), vitality (1 item), social function (1 item), role emotional (2 items), and mental health (2 items). All summary scores were ranged from 0 to 100. A standard Z-score was assigned to each subscale, and the scoring formula was “(actual score minus the lowest possible score of the subscale)/ (the highest score of the subscale minus the lowest score of the subscale) x 100%.“ [32, 33]. A high score reflected good QoL. The Cronbach’s alpha coefficient for PCS and MCS in this study was 0.67 and 0.73, respectively.

### Statistical analysis

First, continuous variables were represented by mean ± standard deviation (SD), while categorical variables were represented by number and percentage (%). To compare the baseline characteristics between the groups with high and low E-R ratio and over-commitment, t-test and χ^2^ test were carried out to analyze the differences in continuous and categorical variables, respectively. Second, the QoL scores were calculated separately for physical and mental health domains, and differences between high and low E-R ratio and over-commitment were determined by t-test. Third, the longitudinal associations of ERI at baseline with changes in QoL with three repeated measures over one year (baseline, 6 months and 12 months) were determined by generalized estimating equations (GEE): the relationships among observations were processed through GEE under the conditions involving the same subjects [34, 35]. In addition to direct associations of E-R ratio, over-commitment at baseline with QoL across one-year period, an interaction term between E-R ratio and over-commitment was also examined to test the effect modification by over-commitment. Three steps were performed in the GEE regression modeling, model I adjusted for sociodemographic such as age, sex, monthly income, marital status, and education; model II additionally adjusted for behavioral factors such as smoking, alcohol drinking, and BMI (kg/m^2^); and model III was based on model II with additional adjustment for clinical characteristics at baseline such as, family history of stroke NIHSS at admission, and Cardiometabolic diseases [36–38]. The SAS 9.4 (SAS Institute, Cary, North Carolina) was used to analyze the data, alpha was set to 0.05 as the significance level.

## Results

### Characteristics of the participants at baseline

As shown in the Tables 1, 103 people who met the eligibility criteria were enrolled, and all of them were invited to participate in the study between December 2020 and August 2022. An analytic sample of 103 patients (68 males and 35 females; mean age 46.8 ± 9.2 years; age range 21–59 years) agreed at baseline and provided complete information data (participation rate = 100%). Age, sex, marital status, monthly income, education level, BMI, current smokers, alcohol drinkers, family history of stroke, NIHSS at admission, and cardiometabolic diseases were not significantly different between the high and low E-R ratio. There were, however, significant differences in male sex, higher monthly income, lower education level, and higher BMI among those with high over-commitment, as shown in Table 1.

**Table 1:** Sociodemographic and clinical characteristics at baseline (n = 103)

		E-R ratio		p value	Over-commitment		p value
		Low (n = 80)	High (n = 23)		Low (n = 69)	High (n = 34)	
Age (years)		47.56 ± 8.82	44.30 ± 10.34	0.18	47.74 ± 8.28	45.00 ± 10.81	0.20
Male, n (%)		54 (67.50)	14 (60.87)	0.55	51 (73.91)	17 (50.00)	0.02
Marital status, n (%)	Single	11 (13.75)	4 (17.39)	0.20	9 (13.04)	6 (17.65)	0.82
	Married	66 (82.50)	16 (69.57)		56 (81.16)	26 (76.47)	
	Divorced/Widowed	3 (3.75)	3 (13.04)		4 (5.80)	2 (5.88)	
Monthly Income (Thai bath)		16954.38 ± 9190.60	15478.26 ± 9418.89	0.51	14814.49 ± 7214.53	20298.53 ± 11598.32	0.02
Education level, n (%)	Less than Bachelor’s degree	15 (18.75)	2 (8.70)	0.25	7 (10.14)	10 (29.41)	0.01
	Bachelor degree or more	65 (81.25)	21 (91.30)		62 (89.86)	24 (70.59)	
Current smokers, n (%)		23 (28.75)	8 (34.78)	0.58	23 (33.33)	8 (23.53)	0.31
Alcohol drinkers, n (%)		38 (47.50)	16 (69.57)	0.06	38 (55.07)	16 (47.06)	0.44
BMI (Kg/m^2^)		24.06 ± 3.72	25.05 ± 4.07	0.30	23.43 ± 3.07	26.00 ± 4.55	0.005
Family history of stroke, n (%)		7 (8.75)	4 (17.39)	0.24	6 (8.70)	5 (14.71)	0.35
Cardiometabolic disease, n (%)		17 (21.25)	5 (21.74)	0.96	12 (17.39)	10 (29.41)	0.16
NIHSS at admission		4.93 ± 3.88	6.74 ± 4.40	0.08	5.25 ± 4.17	5.50 ± 3.85	0.76

Continuous variables were shown as mean ± SD, while categorical variables were shown as frequencies and percentages (n, %). Differences in continuous and categorical variables were examined by a t-test and a chi-squared test, respectively. NIHSS: national institutes of health stroke scale; BMI: body mass index (Kg/m^2^)

### Change of QoL scores over one year after stroke

The QoL scores including SF-12v2 physical health and mental health over 1 year are presented in Table 2. For the SF-12v2 physical health score over 1 year after the stroke, the differences between low and high E-R ratio, and over-commitment were not significant. Regarding the SF-12v2 mental health score, significant differences were observed between low and high over-commitment during the follow-up at 6 months and 12 months, such that individuals with high over-commitment had much lower mental health score, whereas there was no significant difference between low and high E-R ratio during the follow-up period.

**Table 2:** Quality of Life scores over one year

		E-R ratio		p value	Over-commitment		p value
		Low (n = 80)	High (n = 23)		Low (n = 69)	High (n = 34)	
SF12 v2 physical health	Baseline	47.81 ± 16.17	48.91 ± 15.95	0.77	47.58 ± 15.98	49.02 ± 16.38	0.67
	6 months	59.29 ± 27.87	50.00 ± 17.27	0.06	59.19 ± 27.20	53.03 ± 23.39	0.24
	12 months	66.00 ± 24.19	66.45 ± 18.19	0.93	68.45 ± 23.85	61.29 ± 20.76	0.14
SF12 v2 mental health	Baseline	51.12 ± 15.74	46.00 ± 14.82	0.16	51.33 ± 16.85	47.21 ± 12.53	0.17
	6 months	42.80 ± 24.47	40.80 ± 22.42	0.72	45.70 ± 24.68	35.43 ± 20.97	0.03
	12 months	74.43 ± 20.96	72.25 ± 18.30	0.66	78.07 ± 19.02	65.69 ± 20.83	0.007

SF12v2: Short Form-12 questionnaire; E-R ratio: Effort-Reward ratio

Differences in continuous variables were examined by a t-test

### Association of effort-reward imbalance at baseline and changes in QoL one year after stroke

As shown in Table 3, E-R ratio at baseline was not significantly associated with SF-12v2 physical health and mental health scores (all p > 0.05). High over-commitment at baseline was significantly associated with longitudinal worsening scores of SF-12v2 mental health over 1 year after adjustment for sociodemographic, behavioral, and clinical characteristics (coefficient = -8.50; 95% CI = [-13.79, -3.20], all p < 0.05), but was not associated with the SF-12v2 physical health score (all p > 0.05). Furthermore, interaction between E-R ratio and over-commitment on QoL was not significant (all p > 0.05).

**Table 3:** The regression coefficients and 95%CIs of repeated measures of QoL parameters (SF-12) during one-year follow-up by E-R ratio and over-commitment at baseline

		Model I		Model II		Model III	
		Coefficient (95% CIs)	p value	Coefficient (95% CIs)	p value	Coefficient (95% CIs)	p value
SF12 v2 physical health							
E-R ratio	Low	0.00		0.00		0.00	
	High	-0.33 (-5.92, 5.26)	0.91	0.45 (-5.27, 6.16)	0.88	2.53 (-2.93, 7.98)	0.36
Over-commitment	Low	0.00		0.00		0.00	
	High	-4.93 (-10.27, 0.41)	0.07	-3.03 (-8.19, 2.14)	0.25	-1.42 (-5.78, 2.94)	0.52
*P value for interaction*			0.14		0.13		0.38
SF12 v2 mental health							
E-R ratio	Low	0.00		0.00		0.00	
	High	-0.17 (-7.18, 6.84)	0.96	0.39 (-6.61, 7.39)	0.91	2.41 (-4.17, 9.01)	0.47
Over-commitment	Low	0.00		0.00		0.00	
	High	-11.59 (-17.49, -5.68)	0.0001	-9.75 (-16.00, -3.49)	0.002	-8.50 (-13.79, -3.20)	0.002
*P value for interaction*			0.54		0.58		0.43

SF12v2: Short Form-12 questionnaire; E-R ratio: Effort-Reward ration; CIs: confidence intervals; NIHSS: national institutes of health stroke scale; BMI: body mass index (Kg/m2)

Model I: adjusted for sociodemographic at baseline (age, sex, marital status, income, and education)

Model II: Model I + additionally adjusted for behavioral factors at baseline (smoking, alcohol drinking, and BMI)

Model III: Model II + additionally adjusted for clinical characteristics at baseline (family history of stroke, cardiometabolic diseases, and NIHSS at admission)

## Discussion

The purpose of this study was to examine longitudinal associations of work stress in terms of ERI with QoL over a year in Thai workers after a stroke. To the best of our knowledge, this is the first study to consider long-term quality of life and work stress after stroke. Our study hypotheses were only partially supported, as we did not observe direct effects of E-R ratio on QoL, nor an effect modification by over-commitment. However, direct effects of over-commitment on QoL, particularly mental health, were obvious.

According to Siegrist’s ERI model, the first hypothesis is that the imbalance between effort and reward (E-R ratio) has a direct effect on adverse health outcomes [16]. As noted in the introduction, two longitudinal studies among patients with coronary heart disease supported this hypothesis, that E-R ratio at baseline was associated with worsening QoL after one year [18, 19]. However, in our longitudinal study among workers after stroke, this hypothesis was not supported. Similarly, two lab-based and cohort studies did not observe this direct effect of the extrinsic model component on cardiovascular outcomes [39, 40]. In contrast, two case-control studies suggested that E-R ratio was associated with elevated stroke risk [14, 15]. To interpret these discrepancies, we may need to consider that working-age stroke patients in Thailand may have perceptions of what constitutes effort and reward at work that differ from other groups [41]. This also raises the question of appropriate applicability of the E-R ratio in this population. Alternatively, other factors such as physical therapy or medication use may have had a greater impact on QoL among workers after stroke [42]. This latter interpretation is reinforced by strong immediate perceptions after stroke, in particular, worries about disability, with a significant impact on economics [1], loneliness, fear of stroke recurrence [43], life role reduction, negative body image, impaired self-esteem, and loss of employment [44, 45]. Thus, the baseline E-R ratio may not reflect the urgent psychosocial burden among workers who just experienced a life-threatening disease, such as stroke. As indicated by a longitudinal study, that E-R ratio was continuously increased after cardiovascular events, indicating dynamics of changes in work stress before and after onset of a major disease [46].

As for the second hypothesis of ERI, over-commitment, a motivational pattern of people’s coping with stress at work, has a direct effect on negative health, due to the excessive engagement and desire to be in control that render over-committed highly vulnerable to mental stress. In addition, it is also hypothesized that over-commitment could moderate the associations of E-R ratio with outcomes, i.e., effect of E-R ratio on health would be stronger among workers with high over-commitment [47]. The findings of our study supported the second hypothesis, but did not support the third hypothesis given the fact that E-R ratio at baseline was not directly associated with the QoL during the follow-up. Notably, the results showed that high over-commitment at baseline was related to worsening mental health over time, and these effects was independent of sociodemographic factors, behavioral factors, and clinical characteristics. This is in line with previous epidemiological studies with a stronger relationship between over-commitment and mental health, as compared to physical health [48, 49]. Specifically, over-commitment characteristics, such as exerting extreme effort at work and being unable to withdraw from it, demonstrated a direct impact on mental health and quality of life in our study, which could be explained by the fact that Thai workers are under high psychological stress [50, 51] and suffer from negative health effects due to occupational psychological hazards [24]. Moreover, over-commitment seems to directly influence mental health and to induce emotional problems under stressful environment [52, 53]. Clinically, stroke patients frequently experience psycho-emotional difficulties [54], have cognitive and emotional symptoms after their first stroke [55], often persisting over a year [56, 57]. It has been suggested that such emotional patterns and psychological dynamics could activate immune responses, which is a potential mechanism to lead to poor adaptive processes in individuals [58]. As a result, workers who had a stroke are more likely to struggle with disease-related personal and emotional issues, given an unhealthy coping style in terms of over-commitment, such that intrinsic stress would increase the susceptibility to poor mental health outcomes. Within the one-year period of follow-up of this study, a pattern of negative associations between over-commitment and physical health was also observed though not reaching the level of significance.

It is interesting to demonstrate the trajectories of the two components of QoL. Physical health was steadily improved over the year, implying that patients were actually aware of their recovery related to walk, work, and other activities. Furthermore, most participants in this study were diagnosed with a minor stroke. According to clinical observations, minor stroke resulted in good functional status in young adults [59]. On the other hand, the mental health was decreased after six months and then increased at the end of one year. This pattern suggests an elevated fear of disability during the acute stage after stroke onset [60], when patients were most concerned about the uncertainty of disability severity, role limitation, and even their economic future [45]. In addition, the impact of the COVID-19 pandemic on mental health should not be overlooked, as emotional stability was deteriorating and active coping was lacking during this particular period [61]. Contrast to the acute phase, at the twelfth month of follow-up, mental health was greatly improved, in accordance an evident recovery of their physical health.

Several limitations need to be addressed when interpreting the findings of our study. First, the sample size of the study was small, which may limit the generalizability of the results to a larger population. Future studies with diverse samples from other settings would help improve the external validity of the current results. Second, the information on ERI was collected at baseline only in this study, referring to psychosocial work environment before the onset of stroke. Repeated measures of work stress including E-R ratio after return-to work would be valuable for future study. Third, the study was conducted during the COVID-19 pandemic. However, COVID-19 related stressors, for instance, infection fear, loneliness and anxiety due to social distancing and lockdown policies were not measured in our study. Such unexpected and acute stress experiences may marginalize the influence of chronic work stress, resulting in underestimation of the observed associations. By contrast, these observed associations might be overestimated, given the fact that several variables which are related to QoL in stroke survivors were not available in this report, such as detailed job tasks, work ability, cognitive impairment, incontinence, depression/anxiety, coping strategies, social connectedness and caring responsibilities, etc. [62]. Thus, the interpretation of our results should be done with reservation. Still, this study provides an important insight into the relationship between over-commitment and health outcomes, especially mental health in workers after stroke. Despite these limitations, a couple of strengths of this study deserve to be mentioned. First, this is the first longitudinal study on work stress and stroke in Thailand. During the dramatic transition from rural agricultural economy to urban industrialization, Thai working population have experienced stressful working conditions in a rapidly modernized society, such as migration to economically advanced areas for new employment opportunities, long working hours, unstable contract, and low pay [63]. All these are significant contributors to workers’ psychological problems in this country. It is not surprising that occupational psychological hazards have been found to be linked to negative health outcomes, suggested by preliminary research evidence [24, 26]. It is also striking that stroke occurs more often in young people in Thailand, for instance, the annual morbidity rate was increased from 234 to 313 per 100,000 from 2017 to 2020 in working-age group [25]. Second, with longitudinal research design over one-year period with 3-time repeated measure, our findings tightly follow temporal relationships between risk factors at baseline and outcomes during the follow-up, implying potential a causal link between them. Third, patients were assessed with both physical and mental health outcomes, which provides a comprehensive understanding of the profile of QoL.

There are a couple of important implications, based on findings from our study. Obviously, reducing over-commitment would be one important way to promote QoL, especially mental health, among workers after stroke. For instance, one randomized controlled trial (RCT) with 9-year follow-up [64] showed a remarkable effectiveness of stress management interventions on work stress and mental health in the workplace, based on the ERI model. Using cognitive behavioral therapy and psychodynamic therapy for long-term changes in over-commitment, this intervention improved coping skills against stressful work, reduced negative emotions, and strengthened support from colleagues. Furthermore, a recent RCT demonstrated that a stress management intervention improved over-commitment by changing the perception of the psychosocial work environment and attitudes toward work at the third month after the end of program [65]. Thus, evidence of RCTs highlights both short- and long-term consequences of stress management interventions based on the ERI model on improvement of over-commitment. On the other hand, specifically to workers after stroke, promoting early return to work and maintaining their work ability, improving self-esteem and cognitive function are all important factors to facilitate people’s mental health. In this regard, stroke rehabilitation should be prioritized, as early stroke rehabilitation among younger patients was shown to be effective [66], particularly for improving cognitive ability [67]. Unfortunately, working-age stroke patients in this country received less rehabilitation after discharge [68]. As a result, the occupational setting may play an important role in supporting workers in rehabilitation. As shown by recent workplace interventions, organizations could provide more resources for stroke patients to decrease disabilities [69, 70]. One of promising approaches is to integrate organizational- and individual-level resources to support workers in order to be more effective at reducing mental health problems in the long run [71, 72].

## Conclusions

The study investigated the impact of work stress (measured by the ERI model) on the QoL of workers who had experienced a stroke. Findings demonstrated that high over-commitment was associated with worsening QoL over one year, especially its mental health component. This finding suggests that workers’ personal coping skills and ability to handle work-related problems should be considered, and interventions should aim at reducing work-related over-commitment in order to promote long-term mental health among workers who have experienced a stroke.

## Data Availability

The datasets used and/or analyzed during the current study are available from the corresponding author on reasonable request.
